# Myeloid Toxicity of Cancer Treatment

**Published:** 2012-07-01

**Authors:** Sandra Kurtin

**Affiliations:** From Arizona Cancer Center, Tucson, Arizona

## Abstract

Myelotoxicity is one of the most common treatment-related adverse events for patients receiving systemic antineoplastic therapy or radiotherapy to bone marrow–producing regions. Myeloid cytopenias, including neutropenia, thrombocytopenia, and anemia, are the most common manifestations of treatment-related myelotoxicity and one of the most common reasons for dose modifications, dose delays, or discontinuation of therapy, potentially limiting therapeutic benefit. Risk factors for myelotoxicity can be broadly categorized into three types: disease-related, host-related, and treatment- related. Familiarity with factors predictive of high-risk febrile neutropenia, bleeding due to thrombocytopenia, and cardiopulmonary compromise due to anemia will provide the advanced practitioner (AP) in oncology with critical tools for rapid identification of patients at risk, prompt implementation of established guidelines for management, and avoidance of clinical deterioration. The AP in oncology is often the primary point of contact for management of cytopenias, including administration of myeloid growth factors, transfusion of blood products, and management of acute events such as neutropenic fevers. Each of these interventions requires familiarity with the risk and benefits of treatment. This article will review the physiology of the bone marrow, risk factors for cytopenias, and current guidelines and recommendations for prevention and treatment of myeloid toxicity of cancer treatment.

Myelotoxicity is one of the most common treatment-related adverse events for patients receiving systemic antineoplastic therapy or radiotherapy to bone marrow–producing regions. Myeloid cytopenias—including neutropenia, thrombocytopenia, and anemia—are the most frequently seen manifestations of treatment-related myelotoxicity and one of the most common reasons for dose modifications, dose delays, or discontinuation of therapy, potentially limiting therapeutic benefit. Lymphopenia, although less common, presents unique challenges and may place the patient at increased risk for opportunistic and often life-threatening infections. Proactive management of cytopenias can improve treatment tolerance and treatment outcomes. An understanding of the physiology of the bone marrow, normal hematopoiesis, risk factors for treatment-related cytopenias, strategies for minimizing serious adverse events (AEs), and adaptation and consistent application of these concepts for individual patient populations will limit the severity of hematologic AEs and improve treatment outcomes.

The advanced practitioner (AP) in oncology is often the primary point of contact for management of cytopenias, including administration of myeloid growth factors, transfusion of blood products, and management of acute events such as neutropenic fever. The American Society of Clinical Oncology (ASCO), the National Comprehensive Cancer Network (NCCN), the American Society of Hematology (ASH), and the Multinational Association for Supportive Care in Cancer (MASCC) have published recommendations or guidelines for the management of treatment-related cytopenias. The US Food and Drug Administration (FDA) and the American Association of Blood Banks (AABB) have established guidelines for the administration of hematopoietic growth factors and blood products. Familiarity with these guidelines and recommendations, together with a working knowledge of common disease- and treatment-related risk factors, will provide a sound foundation for effective management of treatment-related myelotoxicity.

This article will focus on the clinical management of treatment-related myeloid cytopenias, including current guidelines and recommendations from the societies and associations noted above. Lymphopenia and the management of common infectious complications were previously discussed in *JADPRO*'s series of articles on treatment-related adverse events (Wood & Payne, 2011).

## Bone Marrow Physiology and Normal Hematopoiesis

**Figure 1:**
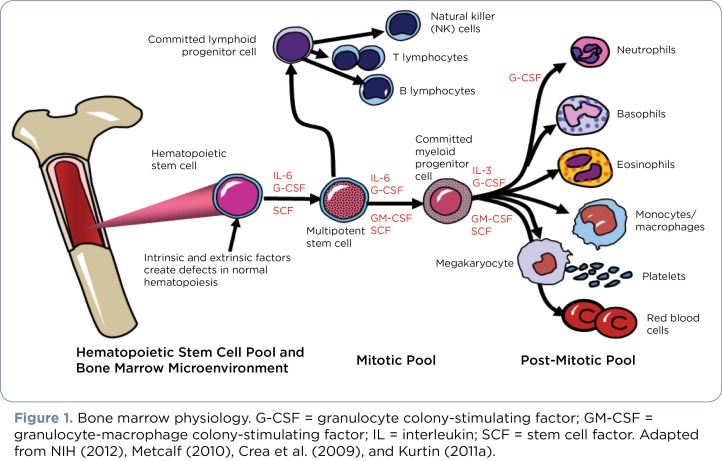
Bone marrow physiology. G-CSF = granulocyte colony-stimulating factor; GM-CSF = granulocyte-macrophage cology-stimulating factor; IL = interleukin; SCF = stem cell factor. Adapted from NIH (2012), Metcalf (2010), Crea et al. (2009), and Kurtin (2011a).

The bone marrow is the primary source for development of the components of blood (hematopoiesis), including myeloid and lymphoid progenitor cells (Figure 1). Hematopoiesis occurs primarily in the axial skeleton, with the majority of production taking place in the pelvis (70%–72%), the long bones such as the femurs, the skull, the sternum, the ribs, and vertebral bodies (Gatter, Natkunam, & Brown, 2008). Extramedullary hematopoiesis, or production of the elements of blood outside the bone marrow, may occur in the spleen and other accessory sites in selected disease states such as the myeloproliferative disorders and chronic leukemia (Kurtin, 2011b). The bone marrow comprises trabecular bone, stromal elements, hematopoietic cells, and elements of the bone marrow microenvironment (Table 1). Each of these elements plays a role in normal or abnormal hematopoiesis and represents a different percentage of the bone marrow space, in part determined by age and underlying disease.

**Table 1:**
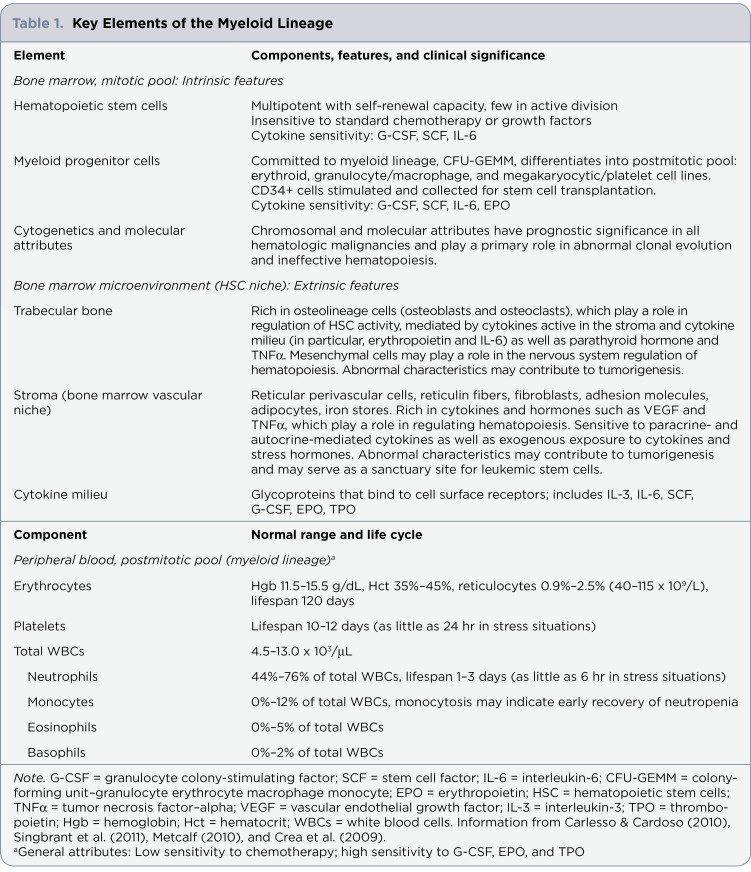
Key Elements of the Myeloid Lineage

Normal myeloid hematopoietic progenitor cells arise from multipotent hematopoietic stem cells (HSCs) that are capable of self-renewal, allowing for continuous replacement of granulocytes, macrophages, and erythrocytes. The majority of HSCs remain noncycling, with only a few active at any time for release of these early progenitor cells (Crea et al., 2009). In the presence of more severe hematopoietic stress, such as bleeding, infection, or chemotherapy exposure, a greater number of HSCs are activated to increase progenitor production (Crea et al., 2009). The normal life cycle for each cell type varies. However, the continuous renewal and life cycle of the myeloid lineage is rapid compared with other tissue cell lines, playing a primary role in susceptibility to the effects of chemotherapeutic agents (Gatter et al., 2008; Maxwell & Maher, 1992).

The capacity for self-renewal is regulated by a number of intrinsic and extrinsic factors, including genetic and molecular mechanisms, cytokines, and signaling pathways (Corey et al., 2007; Bejar, Levine, & Ebert, 2011; Kurtin, 2011a); see Table 1. The process of apoptosis plays a primary role in regulating the "on/off" mechanism of the self-renewal process. Defects in each cell line and in the microenvironment, including apoptosis, play a key role in the pathobiology of hematologic malignancies and contribute to susceptibility to cytopenias, the capacity for hematopoietic recovery, and the abnormal findings in the bone marrow or peripheral blood (Gatter et al., 2008; Carlesso & Cardoso, 2010; Crea et al., 2009). Patients receiving antineoplastic therapies for solid tumors may develop treatment-induced abnormalities, causing similar changes and contributing to cytopenias, although the severity and duration of cytopenias are generally less severe.

The concept of the stem cell niche, highly specialized bone marrow compartments with intricate regulatory processes driving stem cell development and maturation, is an evolving idea in the understanding of normal and malignant hematopoiesis (Carlesso & Cardoso, 2010). These discoveries have elucidated new areas for investigating the prognostic significance of cytopenias at diagnosis and during treatment and the potential for off-target effects of growth factor administration.

Clinical evaluation of bone marrow function relies on analysis of peripheral blood (postmitotic pool) and a bone marrow biopsy and aspirate (mitotic pool/progenitor pool); see Figure 1 and Table 1. A complete blood count with differential and platelet count is the most common method for analysis of the postmitotic pool and will reflect the end results of bone marrow production with any secondary effects of the host. Bone marrow analysis is required for the diagnosis and risk analysis for hematopoietic malignancies, including cytogenetic and molecular information necessary for prognostication. The most common site for obtaining bone marrow samples is the posterior iliac crest, due to the accessibility, relative low risk, and bone marrow–producing capacity. The anterior iliac crest may also be used for sampling, although this is generally more difficult due to the architecture, accessibility, and depth of the cortical bone.

Bone marrow aspirates may be obtained from the sternum using a specialized needle with a shield if there are contraindications for pelvic biopsies, such as for those patients who are intubated and are difficult to turn, for those with a fractured pelvis, or for patients who have had extensive radiotherapy to the pelvis, which may limit the diagnostic value of the specimen. Core biopsies may not be obtained from the sternum due to a high risk of sternal fracture and penetration of surrounding structures with potential for fatal hemorrhage, limiting the diagnostic evaluation. Bone marrow evaluation is performed infrequently in patients with solid tumors unless there are concerns for abnormal findings in the peripheral blood. Adequate bone marrow samples are necessary to provide a complete analysis of the mitotic pool, postmitotic pool, and disease attributes including genetic or molecular features. A summary of the key elements of the bone marrow with normal variants and life cycles for individual cell lines is included in Table 1.

## Risk Factors for Myelotoxicity

Risk factors for myelotoxicity can be broadly categorized into three basic types: disease-related, host-related, and treatment-related. Disease-related factors can be further divided into solid tumors vs. hematologic malignancies. The incidence, severity, and duration of myeloid cytopenias are greatest in the hematologic malignancies. Cytopenias may be present at the time of diagnosis as a result of abnormal cellular development or bone marrow infiltration. In some cases, such as chronic lymphocytic leukemia, autoimmune processes such as hemolytic anemia or idiopathic thrombocytopenia purpura may be present at diagnosis, contributing to the abrupt onset or severity of anemia or thrombocytopenia. Patients with massive splenomegaly due to acute or chronic lymphocytic leukemia or myeloproliferative disorders may present with thrombocytopenia due to splenic sequestration or peripheral destruction of platelets. Thus, evaluation of tumor burden at the time of diagnosis in patients with hematologic malignancies is a critical first step in estimating the risk for cytopenias with initiation of treatment. Furthermore, determining the extent of risk will assist in setting the expectations for both the patient and the providers as treatment is initiated.

It is not uncommon to expect moderate to severe cytopenias in the early phases of treatment for hematologic malignancies, as the primary target for treatment resides in the bone marrow, and both normal and abnormal cells will be affected. Understanding the concepts of expected cytopenias, cytopenias getting worse before they get better, and sustained moderate but asymptomatic cytopenias is critical for the AP in oncology managing patients with hematologic malignancies (Kurtin, Demakos, Hayden, & Boglione, 2012; Kurtin, 2011a). Treatment-related cytopenias are much less common in patients with solid tumors. However, these patients may also experience moderate to severe myeloid cytopenias as a result of bone marrow infiltration by tumor, radiation to bone marrow–producing sites, and other treatment- or host-related factors.

The bone marrow’s capacity to recover is an important consideration in all patients. Patients with limited cellularity, extensive bone marrow fibrosis, underlying aplasias, or treatment-related secondary malignancies have an impaired ability to recover normal hematopoiesis. In most cases, the only option for restoration of normal hematopoiesis is an allogeneic hematopoietic stem cell transplant, which is limited primarily to patients with hematologic malignancies who meet stringent transplant eligibility criteria, including adequate organ function, a suitable donor, and the availability of a consistent caregiver.

## Host-Related Factors

In addition to disease-related factors, selected attributes of the individual patient may increase the risk of treatment-related cytopenias (Table 2). Hematopoietic senescence is common in older patients due to the normal functional decline of the bone marrow with increasing age (Kurtin, 2010). The bone marrow of an older adult is generally less cellular (estimated as bone marrow cellularity % = 100 - patient age in years) with a higher fat content, increasing the susceptibility to cytopenias. Older patients more often experience compromised renal or hepatic function, which may contribute to cytopenias due to impaired metabolism of chemotherapeutic agents (Scripture & Figg, 2006; Kurtin, 2010). Dose modification is required for selected chemotherapeutic agents in the instance of renal or hepatic impairment to limit the severity of AEs, including cytopenias. Similarly, comorbid conditions and associated medications may contribute to decreased bone marrow function or increased risk of drug interactions and treatment-related AEs (Carreca & Balducci, 2009; Scripture & Figg, 2006).

**Table 2:**
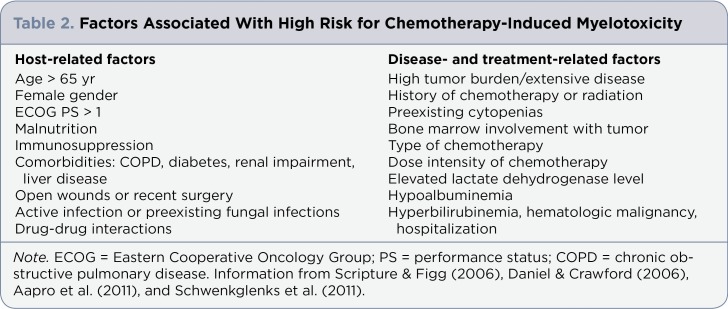
Factors Associated With High Risk for Chemotherapy-Induced Myelotoxicity

Certain drugs are known to be associated with cytopenias independent of bone marrow function, including immunosuppressive agents, anti-inflammatory medications, and antibiotics. Careful review of medications that may contribute to cytopenias is necessary to limit the severity of AEs and to avoid unnecessary dose modification of antineoplastic therapies. Malnutrition affects many cellular processes, including hematopoiesis. Decreased serum albumin levels may indicate an increased risk for treatment-associated AEs and are included in the risk analysis for many hematologic malignancies (Kurtin, 2010; Greipp et al., 2005).

## Treatment-Related Myelotoxicity

Chemotherapy-induced myelosuppression is the most common dose-limiting AE for patients receiving cancer treatment. The incidence, severity, and duration of myelosuppression vary by drug, dependent on pharmacokinetic variables of dose, frequency, route of administration, absorption, distribution, metabolism, and excretion (Undevia, Gomez-Abuin, & Ratain, 2005). Each antineoplastic agent varies with respect to the onset and duration of cytopenias. In general, cytopenias are dose dependent, so dose reductions or delays may be effective in minimizing the severity of cytopenias but may also limit the therapeutic potential of treatment. Dose-intensity and combination therapies are the most common approach used in cancer treatment regimens with the intent to exploit different mechanisms of action and vulnerabilities of the tumor while balancing toxicity profiles. However, increased doses and combination therapies frequently increase the potential for cytopenias (Crawford et al., 2011; Smith, 2006). Several guidelines and recommendations have been proposed by international oncology organizations to estimate the risk for selected cytopenias and to provide evidence-based treatment guidelines (Table 3).

**Table 3:**
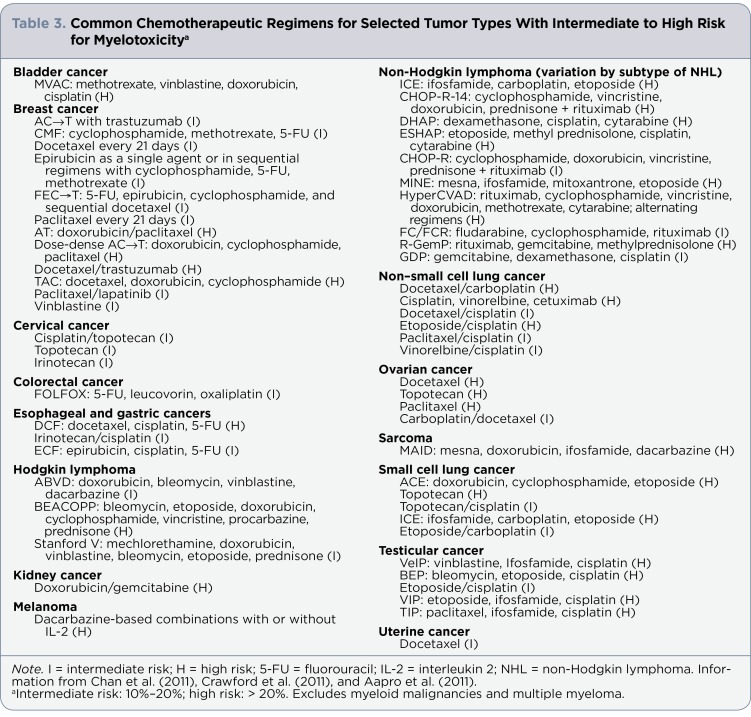
Common Chemotherapeutic Regimens for Selected Tumor Types With Intermediate to High Risk for Myelotoxicity^3^

The combination of chemotherapy and radiation therapy is commonly used to treat esophageal, gastric, head and neck, rectal, anal, and pancreatic tumors. Each of these disease states may include areas of bone marrow–producing regions in the radiation field, increasing the potential for more severe and sustained cytopenias, complicating the delivery of systemic therapy during or following chemoradiotherapy (Chan et al., 2011; Miyoshi et al., 2009). Administration of myeloid growth factors during radiation therapy is generally avoided in these instances, as these agents promote cell division, increasing the potential myelosuppressive effect. Patients receiving treatment over extended periods of time, such as those with metastatic disease, may develop cumulative myelotoxicity and are at risk for secondary malignancies including acute myeloid leukemia (AML) and myelodysplastic syndromes (MDS). The onset of AML and MDS varies according to the agents administered, with early onset (within 3 years) for patients receiving topoisomerase II inhibitors such as etoposide, teniposide, topotecan, and doxorubicin, and late onset (5 to 10 years) for patients receiving radiation or therapeutic alkylators such as cyclophosphamide (Sekeres, 2011; Kurtin, 2011b). Chromosome 5 or 7 abnormalities are most common in these patients and are associated with a poor prognosis.

The late onset of cytopenias, progressive cytopenias unexplained by ongoing treatment, the presence of circulating blasts, or the onset of pancytopenia in cancer survivors will require a bone marrow biopsy and aspirate to further characterize the cause. Specimens should be sent for hematopathology, flow cytometry, and cytogenetic analysis to provide the necessary diagnostic information.

## Clinical Implications of Neutropenia

Chemotherapy-induced neutropenia (CIN) is one of the most common dose-limiting toxicities associated with systemic treatment for cancer due to cytotoxic effects to the rapidly dividing neutrophils, as well as damage to elements of the stroma and cytokine milieu. Mature granulocytes, including neutrophils, have a lifespan of 1 to 3 days, thus they have a rapid mitotic rate and greater susceptibility to cytotoxic damage than other myeloid cell lines with longer lifespans (platelets ~10–12 days, erythrocytes ~120 days; Crea et al., 2009). The onset and duration of neutropenia vary widely by agent, dose, frequency of dosing, and host-related factors previously discussed. Neutrophil precursors are more prevalent than erythroid or platelet cell lines, accounting for more than 50% of the hematopoietic cells in the bone marrow and postmitotic pool, with only 2% in circulation and 3% in the spleen or vasculature (Crea et al., 2009).

The actively dividing cells in the mitotic pool and postmitotic maturation pool are the most sensitive to the effects of chemotherapy, whereas mature and fully differentiated cells in the peripheral blood are less sensitive. The degree of sensitivity for different cells in the maturation process, together with the lifespan of each cell line, helps to explain the time of onset and recovery of cytopenias and the principles of growth factor administration for treatment.

The presence of neutropenia predisposes patients to infection. The severity and duration of neutropenia, together with host-related factors and secondary effects of the treatment regimen, contribute to the risk of more serious AEs, including neutropenic fevers and bacteremia. The greatest risk of severe CIN, including febrile neutropenia (FN), is in the first cycle of chemotherapy (Klastersky, Awada, Paesmans, & Aoun, 2010; Aapro, Crawford, & Kamioner, 2011; Wingard & Elmongy, 2009). As a result, the prophylactic use of colony-stimulating factors is recommended when the risk of National Cancer Institute Common Terminology Criteria for Adverse Events (CTCAE) grades 3/4 CIN or FN is greater than 20% in the setting of potentially curable disease where dose intensity is necessary for optimal clinical outcomes (Crawford et al., 2011; Wingard & Elmongy, 2009); see Table 3. Additional parameters are suggested for patients with < 20% risk of CTCAE grades 3/4 CIN or FN; see Table 4.

**Table 4:**
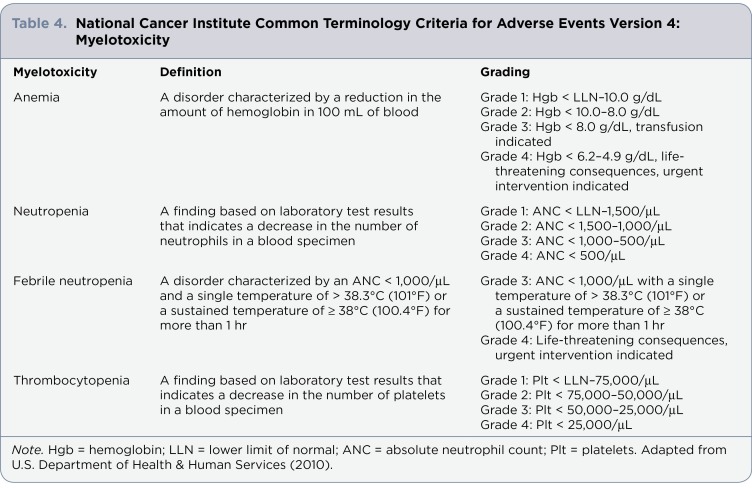
National Cancer Institute Common Terminology Criteria for Adverse Events Version 4: Myelotoxicity

Chan and colleagues (2011) conducted a systematic literature review to evaluate the incidence, patient characteristics, severity, supportive care strategies, and clinical impact of grades 3/4 CIN for patients enrolled in randomized phase III clinical trials evaluating emerging regimens for the treatment of common solid tumors (Chan et al., 2011). A total of 1,522 randomized, controlled trials for breast, lung, ovarian, colorectal, or renal cell carcinomas were evaluated for the inclusion of criteria for reporting, grading, and management of clinically significant CIN. Only 264 of the published trials reported the incidence of grades 3/4 CIN (72%) or FN (53%) overall. The incidence by tumor type for grades 3/4 CIN or FN was most common in the studies for lung (n = 89: 82%, 60%), ovarian (n = 25: 80%, 52%), and breast (n = 73: 70%, 55%) cancer, with fewer cases reported for colorectal (n = 63: 60%, 53%) and renal cell (n = 14: 57%, 21%) cancers.

Only 73% of the trials included descriptions of strategies for management of these events. The most commonly reported strategies for management included dose delays (36%), dose reductions (48%), and granulocyte colony-stimulating factor (G-CSF) administration. The discussion of G-CSF administration in the methods (38%) or results (19%) segment for each published study was rare. The authors note that this as a major limitation for application of the trial results to the general oncology population (Chan et al., 2011). Given the well-established clinical guidelines for the management and prevention of CIN and FN, inclusion of these strategies in the design and reporting for clinical trials for emerging therapies is critical to provide oncology clinicians with the data necessary to safely integrate these newer therapies into clinical practice, limit the severity of AEs, and provide patients with the most effective clinical outcomes.

Patients with FN may deteriorate rapidly. Prompt management of these patients is essential to avoid more severe AEs such as sepsis syndrome, circulatory collapse, acute respiratory failure, or death. Familiarity with factors predictive of high-risk FN will provide the AP in oncology with critical tools for rapid identification of these patients, prompt implementation of established guidelines for management, and avoidance of clinical deterioration (Table 5).

**Table 5:**
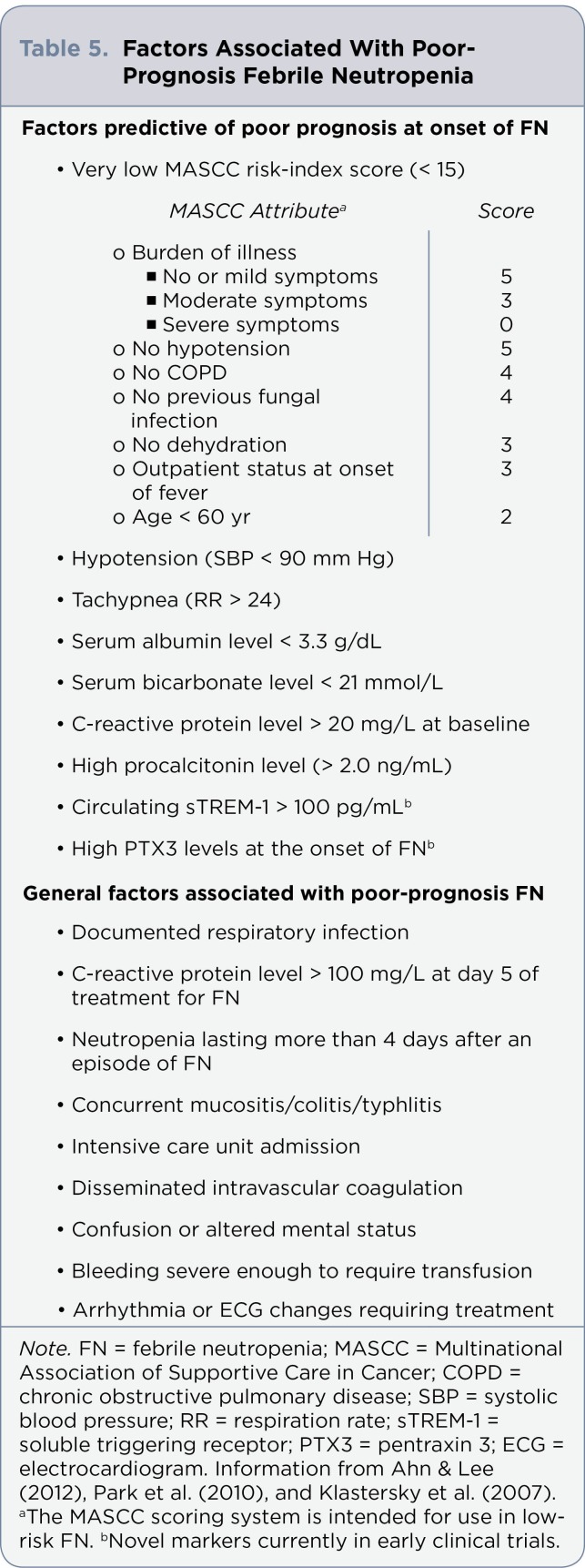
Factors Associated With Poor-Prognosis Febrile Neutropenia

Patients with CIN or FN generally present with fever and/or shaking chills. Low-risk patients may be asymptomatic and found to be neutropenic on routine laboratory evaluation. The general principles of treatment for a patient with grades 3/4 CIN or FN include rapidly assessing the level of risk for deterioration and stabilizing the patient if necessary, while simultaneously obtaining additional laboratory measures, including blood cultures and urinalysis for culture and sensitivity. Prompt implementation of institutional guidelines or standing orders for the management of neutropenic fevers, including antibiotic administration, is critical to prevent more serious complications.

Park and colleagues (2010) evaluated 259 episodes of FN in patients with hematologic malignancies (n = 137; median age 48, range 18–85 years). The most common organisms isolated were gram-negative bacteria (*Escherichia coli* most common, 50 episodes) and gram-positive bacteria (*Staphylococcus aureus* most common, 22 episodes). The respiratory tract (69 episodes, 26.6%), indwelling catheters (22 episodes, 8.5%), and the gastrointestinal tract (20 episodes, 7.7%) were the most common documented sites of infection (Park et al., 2010). Of the 259 episodes evaluated, 43% (107 episodes) were documented as fever of unknown origin.

In a second trial, Klastersky and colleagues (2007) applied the MASCC risk criteria (Table 5) to a population of 2,142 cancer patients with solid tumors and hematologic malignancies receiving chemotherapy to evaluate the incidence of bacteremia in patients experiencing FN. Fifty-eight percent of the patients were considered to be at low risk for complications related to FN. A total of 499 patients (23%; median age 52 years) developed documented bacteremia. Gram-positive bacteremia was most common (57%), with gram-negative (23%) and polymicrobial bacteremia (10%) less common. A MASCC risk score of < 15 was associated with the poorest prognosis (*p* < .001).

These trials emphasize the increased risk for CIN and FN in patients with hematologic malignancies, the common sources of infection, and the most common microbes isolated, providing the AP in oncology with useful information for evaluating risk and identifying treatment strategies.

Once cultures are obtained, the first priority is to administer IV antibiotics based on institutional policy, given variations in common microbial profiles by institution and by region (Klastersky et al., 2011). In the study conducted by Park et al. (2010), the most common first choice of antibiotics administered was cefepime (142 episodes, 55%). The addition of a glycopeptide (e.g., vancomycin) for sustained fevers after 3 days of empiric antibiotics was most common, with the addition of antifungal agents based on suspected fungal etiology (Klastersky et al., 2011).

Factors associated with poor outcomes based on univariate analysis of the 259 FN episodes included age (*p* = .010) and comorbidities such as hypertension (*p* = .012) and liver disease (*p* = .029). The leading cause of death (n = 61 [24%]) was septic shock with multiorgan failure (21 cases) and respiratory failure (9 cases). Patients who developed a fever outside the hospital experienced more serious complications (*p* = .003), emphasizing the need to have established protocols and effective patient and caregiver communication to expedite the management of FN in patients being cared for in the outpatient setting, particularly older patients. Recovery from neutropenia was found to be the most significant factor for survival (*p* < .0001). Additional factors associated with poor-prognosis FN are included in Table 5.

In general, risk analysis based on the concepts previously discussed, incorporation of prophylactic administration of myeloid growth factors in high-risk populations based on consensus guidelines, and strategies for early identification and prompt initiation of treatment of FN are the backbone of managing CIN. Recommendations for the prevention and treatment of CIN and FN are summarized in Table 6.

**Table 6:**
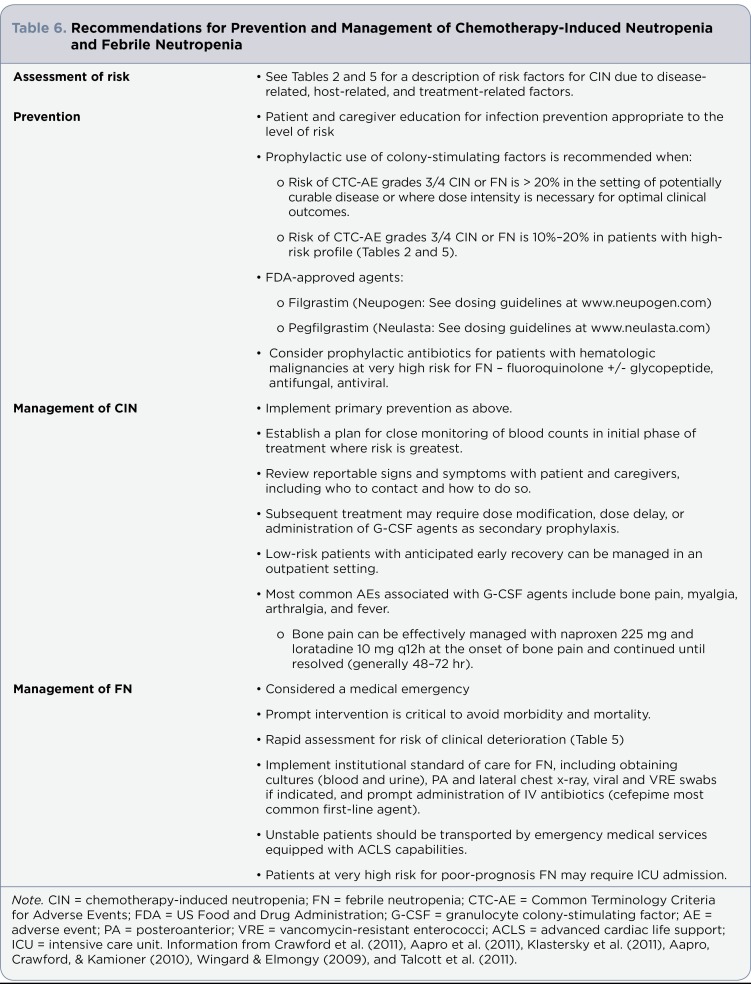
Recommendations for Prevention and Management of Chemotherapy-Induced Neutropenia and Febrile Neutropenia

Once a patient has experienced an episode of grades 3/4 CIN or FN, several additional considerations in continuing treatment come into play. Unlike patients who experience milder CIN (grades 1/2), patients with more severe episodes will require greater scrutiny. The decision to resume treatment is based on the individual patient; his or her risk profile including underlying disease; the treatment regimen including therapeutic intent; and the availability of supportive care including growth factor support, accessibility to the clinical setting, financial resources, and caregiver support. Dose modification and/or dose delays may be required in patients with metastatic or incurable disease who may require treatment over extended periods of time.

The use of G-CSF as secondary prophylaxis has also been studied. Guidelines proposed by ASCO and the NCCN suggest the use of G-CSF in patients receiving mild to moderately myelotoxic chemotherapy with curative intent who have experienced an episode of grades 3/4 CIN or FN (Crawford et al., 2011; Aapro et al., 2011). Myeloid growth factors are not without potential AEs, the most common being bone pain (Table 6). Bone pain associated with G-CSF is generally self-limiting but may be severe (Wingard & Elmongy, 2009). The administration of an anti-inflammatory agent such as naproxen together with an antihistamine such as loratadine at the onset of bone pain and continued for 48 to 72 hours works well to reduce the pain, with little risk of toxicity and minimal cost to the patient.

The use of prophylactic antibiotics remains controversial owing to limited data substantiating their benefit in the general cancer population together with concerns for the development of drug-resistant bacteria. Unlike myeloid growth factors, which have proven efficacy in preventing the incidence and severity of neutropenia, including episodes of FN, prophylactic antibiotics serve to reduce the complications of neutropenic fevers (Wingard & Elmongy, 2009). More recent trials have documented a reduction in the incidence of neutropenic fevers and bacteremia and infection-related mortality with administration of fluoroquinolones (ciprofloxacin and levofloxacin most common) in patients with hematologic or solid tumors (Wingard & Elmongy, 2009; Wood & Payne, 2011). Fluoroquinolones provide excellent coverage against *Pseudomonas aeruginosa* (ciprofloxacin) and *Streptococcus* (levofloxacin).

The concern for antibiotic-associated AEs, additional cost, secondary infection with *Clostridium difficile*, and the emergence of drug-resistant bacteria must be considered. However, prophylactic antibiotics in patients with hematologic malignancies or in patients at high risk for poor-prognosis FN should be considered. Combining prophylactic antibiotics with G-CSF, in particular pegfilgrastim if the schedule permits, may provide the best option for prevention in very high-risk patients (Wingard & Elmongy, 2009).

## Clinical Implications of Anemia

Anemia is a common finding in patients with cancer, with an incidence ranging from 30% to 90% (Rodgers et al., 2012). The causes of anemia in cancer patients vary widely, including metabolic and nutritional causes, chronic disease, renal insufficiency, blood loss, inadequate production due to bone marrow disease, peripheral destruction due to autoimmune disorders, drug-induced red cell aplasia, and chemotherapy-induced anemia (CIA). Anemia is multifactorial in most cancer patients due to the generally older age of the cancer population, the existence of comorbidities with associated medications, and the effects of the malignancy and treatment (Rodgers et al., 2012). Chemotherapy-induced anemia is far less common than CIN, in part due to the difference of the life cycle of red blood cells; however, chemotherapeutic agents may contribute to anemia through disruption of normal hematopoiesis and interference of the cytokine milieu (Crawford et al., 2011).

The primary approach to the patient with anemia is as follows: (1) Establish the underlying cause(s), (2) treat the underlying cause(s), (3) evaluate symptoms of anemia with consideration of individual patient characteristics, and (4) weigh the risks and benefits of each treatment approach. The comprehensive assessment and management of anemia in the patient with cancer is beyond the scope of this article. However, supportive care for those receiving myelotoxic chemotherapy is an essential component of clinical management for these patients, including the treatment of anemia. Beyond the treatment of comorbid conditions and other contributing factors, the primary interventions for CIA include therapeutic transfusion of packed red blood cells (PRBCs) and administration of erythropoiesis-stimulating agents (ESAs; Rodgers et al., 2012).

The FDA, the AABB, and the NCCN have recently revised the guidelines for administration of ESAs and PRBCs based on the specific risks and benefits of each approach (Carson et al., 2012; Vlaar, et al., 2011; Rodgers et al., 2012). Familiarity with these very recent recommendations is critical for the AP to provide informed consent to the patient, to maintain safety, and to ensure compliance with regulatory requirements, including the Risk Evaluation and Mitigation Strategy (REMS) program for erythropoiesis-stimulating agents regulated by the FDA and institutional blood bank requirements (Table 7).

**Table 7:**
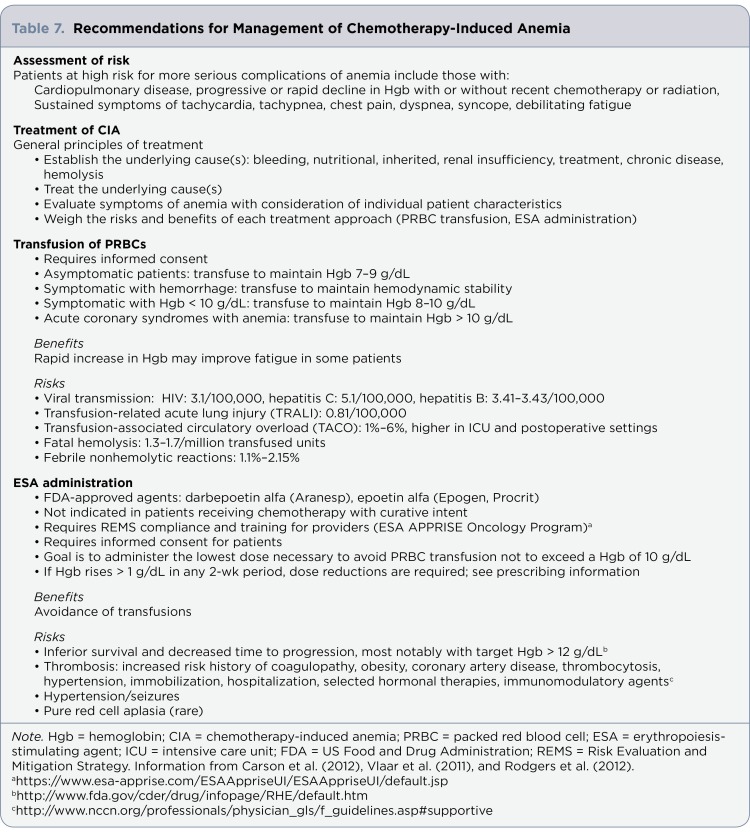
Recommendations for Management of Chemotherapy-Induced Anemia

Consideration of the individual patient characteristics and associated risks of PRBC transfusion and ESA administration is necessary. Thresholds for transfusion in particular will vary according to patient age, comorbidities, underlying disease, expected capacity for hematologic recovery, and ongoing therapy. It must always be kept in mind that the benefits of any transfusion are temporary, and restoration of normal bone marrow function and erythropoiesis is the most desirable goal. As noted in the REMS guidelines, there are specific risks associated with the administration of ESAs, including thrombosis, inferior survival, and decreased time to progression in selected tumor types. Thus, ESAs are reserved for patients receiving chemotherapy where treatment does not have a curative intent (Rodgers et al., 2012).

## Clinical Implications of Thrombocytopenia

Thrombocytopenia and the risk of bleeding present a particular challenge in cancer patients undergoing cancer treatment. Like anemia, the cause may be multifactorial, including underlying disease, comorbidities, associated medications, disease-related destruction, or as a direct effect of treatment on the macrophages in the mitotic pool. Unlike neutropenia or anemia, the use of colony-stimulating factors for chemotherapy-induced thrombocytopenia is not yet FDA approved, in part due to concerns for off-target effects of thrombopoietic agents. The lifespan of a mature platelet in the postmitotic pool may be as little as 24 hours, thus there is a constant need for replacement. Patients with bone marrow disorders, continued peripheral destruction, or sequestration such as with splenomegaly are at particular risk for sustained thrombocytopenia. The risk of bleeding is the greatest concern for these patients. Criteria for therapeutic or prophylactic platelet transfusions, the type of platelets, and volume transfused have been reviewed (Slichter et al., 2010; Triulzi et al., 2012).

The Platelet Dose Study (PLADO), a database analysis of 3,447 hematology/oncology patients who received therapeutic platelet transfusions, found a platelet count of 10,000/µL to be the recommended trigger for prophylactic transfusion, with adaptation for patients with complicating factors, consistent with previous trials and consensus guidelines (Triulzi et al., 2012; Schiffer et al., 2001; Slichter, 2007; Slichter et al., 2010); see Table 8. A platelet count ³ 5,000/µL is thought to be sufficient to maintain endothelial integrity, a key factor in spontaneous bleeding risk. Educating the patient and their caregivers about bleeding precautions and reportable signs and symptoms is critical to avoiding more serious adverse events.

**Table 8:**
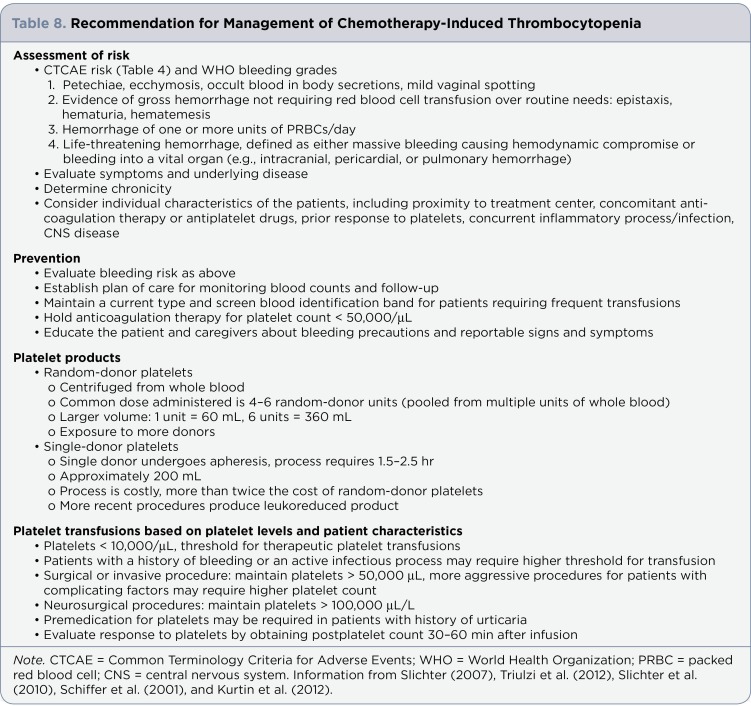
Recommendation for Management of Chemotherapy-Induced Thrombocytopenia

## Summary and Conclusions

Chemotherapy-induced myelotoxicity is a common and potentially life-threatening adverse event for cancer patients. Neutropenia, febrile neutropenia, anemia, and thrombocytopenia are the result of complex processes as a result of the disease, the approach to treatment, and the characteristics of the individual patient. Assessment of individual risk using the criteria described, implementing prevention, monitoring, and treatment strategies, as well as setting expectations for the patient and family are essential to avoid more serious adverse events. Familiarity with the risks and benefits of supportive care measures for the treatment of cytopenias, recent updates to consensus guidelines, recommendations for treatment modification, and contraindications for various supportive care measures will assist the AP in oncology in effectively managing chemotherapy-induced myeloid toxicity.
